# Investigation of potential adverse central nervous system effects after long term oral administration of gadolinium in mice

**DOI:** 10.1371/journal.pone.0231495

**Published:** 2020-04-23

**Authors:** Dominik Nörenberg, Felix Schmidt, Karin Schinke, Thomas Frenzel, Hubertus Pietsch, Armin Giese, Birgit Ertl-Wagner, Johannes Levin

**Affiliations:** 1 Department of Clinical Radiology and Nuclear Medicine, University Medical Center Mannheim, Mannheim, Germany; 2 Department of Radiology, Munich University Hospitals, LMU, Munich, Germany; 3 Munich Center for Neuropathology, Ludwig-Maximilians-University Munich, Munich, Germany; 4 Department of Neurology, Munich University Hospitals, LMU, Munich, Germany; 5 MR and CT Contrast Media Research, Bayer AG, Berlin, Germany; 6 Department of Medical Imaging, The Hospital for Sick Children, University of Toronto, Toronto, Canada; 7 German Center of Neurodegenerative Diseases (DZNE), Munich, Germany; Charite Universitatsmedizin Berlin, GERMANY

## Abstract

**Objectives:**

To examine potential gadolinium (Gd) accumulation in the brain of healthy mice after long-term oral administration of Gd-containing food pellets and to investigate whether Gd leads to adverse central nervous system (CNS) effects, specifically focussing on locomotor impairment in Gd exposed compared to control animals.

**Materials and methods:**

The local Animal Experimental Ethics Committee approved all procedures and applications. Fifteen female C57Bl/6 mice were orally exposed to a daily intake of 0.57 mmol Gd chloride/ kg body weight over a period of 90 weeks from the age of 4 weeks on. Gd-free, but otherwise equivalent experimental diets were given to the control group (N = 13). The animals were monitored daily by animal caretakers regarding any visible signs of distress and evaluated clinically every four weeks for the first 60 weeks and afterwards every two weeks for a better temporal resolution of potential long-term effects regarding impairment of motor performance and loss of body weight. The individual Gd content was measured using mass spectrometry in a sub-cohort of N = 6 mice.

**Results:**

The absolute brain Gd levels of the Gd-exposed mice were significantly increased compared to control mice (0.033± 0.009 vs. 0.006± 0.002 nmol Gd/ g brain tissue). Long-term oral Gd exposure over almost the entire life-span did not lead to adverse CNS effects including locomotor changes (rotarod performance, p = 0.1467) in healthy mice throughout the study period. Gd-exposed mice showed less increased body weight compared to control mice during the study period (p = 0.0423). Histopathological alterations, such as hepatocellular vacuolization due to fatty change in the liver and a loss of nucleated cells in the red pulp of the spleen, were found in peripheral organs of both groups.

**Conclusions:**

Low levels of intracerebral Gd caused by chronic oral exposure over almost the entire life span of mice did not lead to alterations in locomotor abilities in healthy mice throughout the normal aging process.

## Introduction

Gadolinium (Gd) is a rare-earth lanthanides metal with strong paramagnetic properties. According to extensive application of Gd-containing agents in biomedical fields, it will enter the body through some administration routes such as oral or intravenous injections. Nowadays, Gd is widely utilized for various industrial and medical purposes, particularly as intrevanous contrast agent in magnetic resonance imaging (MRI) [1–3]. Gadolinium-based contrast agents (GBCAs) are an essential tool in MRI diagnostics and, until recently, had been generally considered to have an excellent safety profile, aside from the risk of nephrogenic systemic fibrosis (NSF) in patients with end-stage renal failure and very infrequent cases of acute neurotoxicity [4–7]. The history of NSF and GBCAs is well documented [8]. Over recent years it has become apparent that exposure to GBCAs can potentially result in gadolinium deposition within different human tissues or organs (such as bone, liver, kidney) even in the presence of normal renal function [1, 9–12]. Additionally, several recent studies have linked an increase in signal on non-enhanced, T1-weighted MR images in certain areas of the brain to a prior history of GBCA-enhanced MR examinations. Furthermore, accumulation of Gd was observed in brain tissue of small animals and in autopsy studies of humans [10, 11, 13–28]. This is a relatively new and growing field of research primarily driven by concerns regarding unknown and potentially harmful side effects of GBCAs due to brain accumulation. Currently, there is no clear evidence linking Gd and its known brain accumulation with central nervous system (CNS) toxicity or locomotor impairment [9].

Over the last few decades, rapid industrial development and the concomitant increase in the clinical use of GBCAs for medical diagnostics in MRI resulted in a considerable increase of the anthropogenic Gd content in aquatic ecosystems in industrialized regions, thus representing Gd as an emerging environmental contaminant [29, 30]. Prior studies investigating the biodistribution after oral ingestion of Gd-containing nanotubes in rodents found that gadolinium can accumulate in very low concentrations in a range of tissues and organs (skin, bone, liver, kidney, muscle and spleen) [31], however, its brain accumulation was not assessed. Of note, the uptake and distribution of intravenously administered GBCAs differs substantially from those observed after oral exposure to gadolinium salts, and there are no data available assessing brain accumulation after oral Gd exposure. Given the increasing environmental Gd contamination, studies investigating potential adverse CNS effects of intracerebral Gd accumulation after oral exposure are warranted. We therefore aimed to (i) measure the levels of gadolinium present in the brain of mice after life-long oral exposure and (ii) to investigate associated adverse CNS effects with focus on impairment of locomotor abilities due to chronic oral exposure.

## Materials and methods

### Animals and gadolinium administration

As part of a larger study, a cohort of 28 in-house bred female C57Bl/6 mice were used to investigate the effects of chronic oral Gd intake (N = 15) compared to reference diet (N = 13) on general health, as indicated by impact on gain of body weight, on locomotor activity in the rota rod motor performance test, and on histopathological alterations in the brain and peripheral organs, respectively. As mouse tissue was used for different analytical purposes in context of a larger study, histopathological analysis as well as measurements of Gd accumulation in the brain were performed on available subgroups of N = 9 or 8 mice (histopathological examination), and N = 6 per group (analysis of Gd brain levels). Analysis of progression of body weight, as well as motor performance was conducted based on the data of the whole group (N = 15 or 13) to strengthen the power of the statistical analysis. The Local Animal Experimental Ethics Committee of the government of Upper Bavaria approved all procedures and applications (reference number: 55.2-1-54-2531-32-08). Mice were housed in a specific pathogen-free (SPF) animal facility with free access to food and water, the light and dark cycle was 12 h/12 h and the temperature was kept constant at 22°C. At the age of 4 weeks mice were randomized into two groups. Over a study period of 90 weeks one group (N = 15) received food pellets containing 600 mg Gd-chloride/kg food (Ssniff, Soest, Germany) resulting in a daily intake of approximately 0.57 mmol/ kg body weight, whereas the age-matched control group (N = 13) received experimental diets low in metal ions containing only 60 mg Fe-chloride/ kg food to prevent adverse clinical effects caused by iron deprivation [32]. The animals were monitored daily by animal caretakers regarding any visible signs of distress and evaluated clinically every four weeks for the first 60 weeks and afterwards every two weeks for a better temporal resolution of potential long-term effects. According to approved protocols mice were sacrificed after reaching the maximum observation period of 90 weeks. Mice were sacrificed by cervical dislocation and subsequently submitted to histopathological examination.

### Assessment of locomotor abilities

Beginning at 20 weeks of age until reaching the maximum observation period of 90 weeks, mice were additionally evaluated with standardized locomotor tests. For assessment of motor performance, a rota rod advanced V4.1.1 (TSE Systems, Chesterfield, MO, USA) was used as described previously [33–35]. Briefly, two days prior to the rota rod performance test, mice were trained in three trial runs with an acceleration of the rod from 0 to 30 rpm over 180 s. On the third day, the test consisted of three runs with an acceleration from 0 to 50 rpm over 300 s. The latency between each trial run was at least 40 s. The average time on the rota rod and the best performance out of three runs were evaluated. Runs lasting less than 10 s were defined as invalid and repeated up to a maximum of three repeats per test day. Animals with a best run of less than 20 s would be considered terminally ill and sacrificed.

### Tissue processing and histopathological assessment

For histopathological investigation, the right brain hemisphere as well as the spinal cord and peripheral organs (heart, lung, liver, spleen, kidney, and gastrointestinal tract) were fixed in 4% formalin and embedded in paraffin. 1 μm tissue sections were deparaffinized and stained with haemalum for 10 min (Chroma Waldeck, Muenster, Germany). GFAP-stainings were performed using a polyclonal rabbit antibody (Agilent Technologies, Waldbronn, Germany) 1:2000 for 32 min and a biotinylated secondary antibody swine-anti rabbit (Agilent) 1:150 for 20 min. Sections were washed in lukewarm Millipore water for 10 min, shortly incubated in 70% EtOH and stained in eosin for 2 min (Sigma Aldrich, Taufkirchen, Germany). The left-brain hemisphere of every animal was natively snap-frozen over liquid nitrogen and stored at -80°C until further analysis.

H&E- and GFAP-stained sections of the right brain hemisphere and the spinal cord on five different heights as well as H&E-stained sections of all peripheral organs were used for histopathological examination. Assessment was performed by a single experienced rater blinded to the treatment of the individual animal using an Olympus BX 50 microscop and a UPlanFl 4x/0.13 objective (Olympus, Hamburg, Germany). The rating included appropriate grading of extent or severity of any neoplastic and non-neoplastic morphologic changes in the categories absent, mild, moderate, or severe. These categories were further translated to values ranging from 0 (no pathology) to 3 (severe pathology) to allow for analysis of group effects on histopathological alterations. Therefore, a composite score was calculated by summing up the individual values for histopathological alterations in all organs.

### Measurement of intracerebral Gd-accumulation

Gd levels in the left brain hemisphere of 6 mice of both diet groups were determined by using inductively coupled plasma–mass spectrometry (ICP-MS) randomized and blinded to the treatment group. The tissue samples were homogenized with a scalpel and 3 aliquots of 20 mg were mixed with 50 μl of a 100 nmol/ l solution of Zn(NO3)3 as internal standard. The samples were dried at 90°C under a laminar flow bench. To digest the tissue 50 μl nitric acid (65%) and 30 μl of hydrogen peroxide (25%) were added. The closed samples were heated in a microwave oven for 30 min at 120°C. The clear samples were then diluted with nitric acid (1%, containing 0.01% Triton-X 100). The measurement of Gd was performed on an ICP-MS (Agilent 7900, Agilent) using commercial calibration standards (Merck Suprapur, Merck, Darmstadt, Germany) which all contained 5 nmol Tb/ l as internal standard. Blanks were positioned between the tissue samples to avoid carry over effects. The limit of quantification (LOQ) of this method is 0.1 nmol Gd/ l in the final solution which is equivalent to 0.005 nmol Gd/ g wet tissue.

### Statistical analyses

All statistical analyses were conducted by using GraphPad Prism software (GraphPad Software, La Jolla, USA) and statistical significance was set to p< 0.05.

Mean accumulation of Gd in brain tissue as well as mean composite score of histopathological alterations were analyzed using a two-tailed t-test. Survival of animals was analyzed using the Log-Rank (Mantel-Cox) test. Gain of body weight, as well as motor performance were analyzed using mixed-effect model analyses due to missing values for mice being sacrificed before the end of the study period, and corrected for multiple comparisons by Sidak’s multiple comparisons test.

## Results

### Gadolinium accumulates in the brain of healthy mice after long-term oral exposure of gadolinium chloride

**Fig 1:**
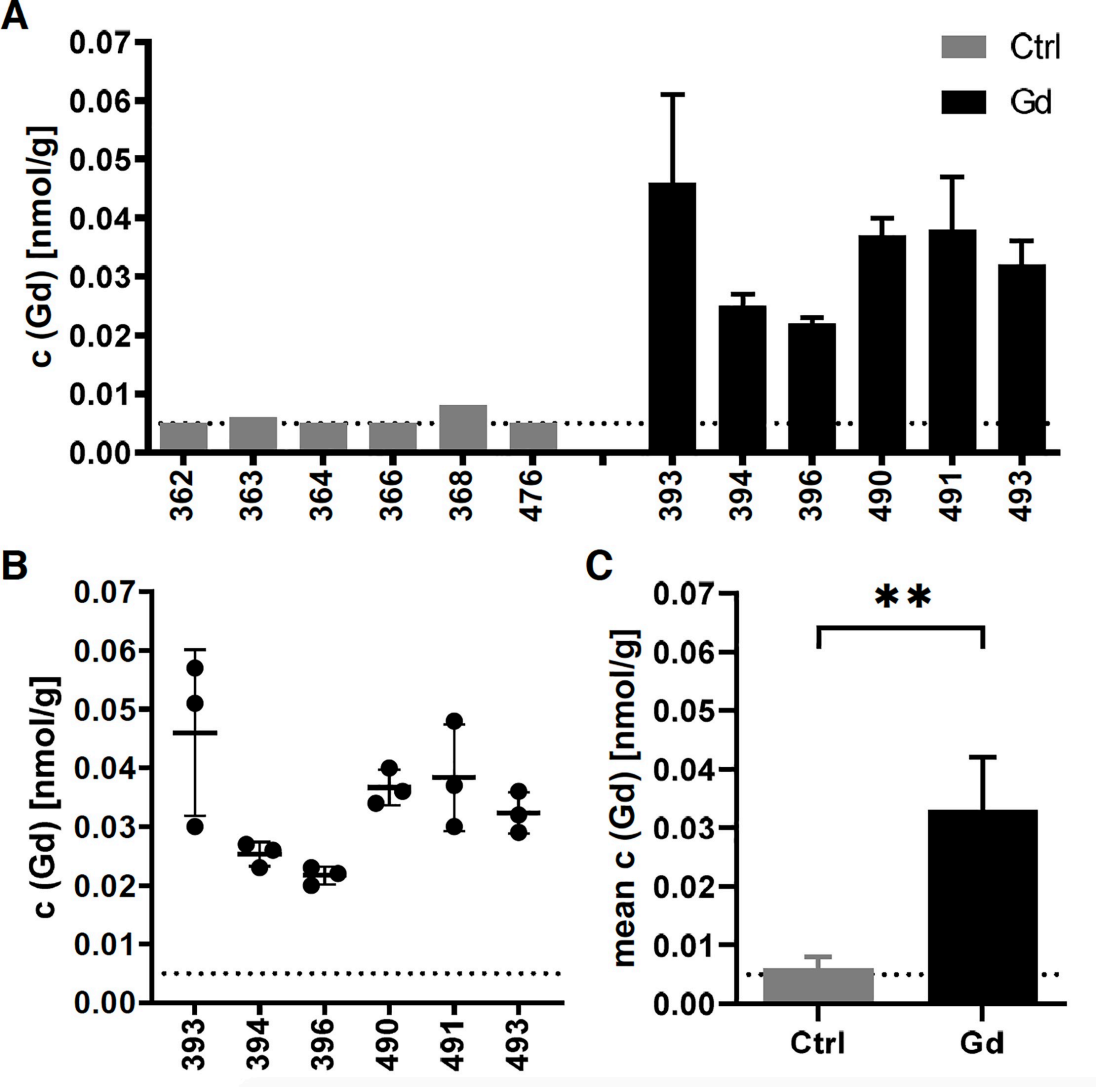
Accumulation of Gd in the brain. Oral exposure to Gd led to detectable levels of Gd in the brain of treated animals (N = 6) compared to control mice (N = 6), where individual values were near or even below the LOQ (0.005 nmol/ g; dotted line) (A). Mean± SD of 3 replicate measurements per individual brain hemisphere of Gd-treated animals (B). Oral exposure to Gd lead to a significantly increased accumulation of 0.033± 0.009 nmol/ g brain tissue compared to 0.006± 0.002 nmol/ g brain tissue in control mice. Mean± SD of 6 brain hemispheres per group (two-tailed t-test, p = 0.0016; see Table 1 for detailed individual information).

Gd-exposed mice showed an average Gd-concentration in the brain of 0.033± 0.009 nmol Gd/ g compared to 0.006±0.002 nmol Gd/ g brain tissue in control mice (Fig 1; mean± SD of 6 brain hemispheres per group; two tailed t-test, p = 0.0016). Of note, all values in reference tissue were near (N = 3), or even below (N = 3) the limit of quantification (LOQ = 0.005 nmol/ g; see Table 1 for detailed individual information).

**Table 1:** Summary of individual measured Gd concentration per gram wet brain tissue.

	ID	age [w]	c (Gd) [nmol/g]	SD	remarks
**Control**	362	94.3	< 0.005	n.a.	all 3 samples < LOQ
	363	94.3	0.006	n.a.	2 of 3 samples < LOQ
	364	94.3	0.005	n.a.	2 of 3 samples < LOQ
	366	94.3	< 0.005	n.a.	all 3 samples < LOQ
	368	94.3	0.008	n.a.	2 of 3 samples < LOQ
	476	94.9	< 0.005	n.a.	all 3 samples < LOQ
	**mean**		**0.006**	**0.002 **	
**Gd-exposure**	393	94.3	0.046	0.015	
	394	90.0	0.025	0.002	
	396	94.3	0.022	0.001	
	490	94.9	0.037	0.003	
	491	94.9	0.038	0.009	
	493	94.9	0.032	0.004	
	**mean**		**0.033**	**0.009**	

Every value represents the average of three replicates. In three reference mice all replicates were below the limit of quantification (0.005 nmol Gd/ g brain tissue).

### Gadolinium exposed animals reveal mild adverse health effects with a decrease in body weight gain, but do not reveal impairment in locomotor behavior throughout the study period

To analyze the effects of oral Gd exposure on clinical outcome we investigated the motor performance, gain of body weight, as well as the survival of Gd-treated compared to control mice.

**Fig 2:**
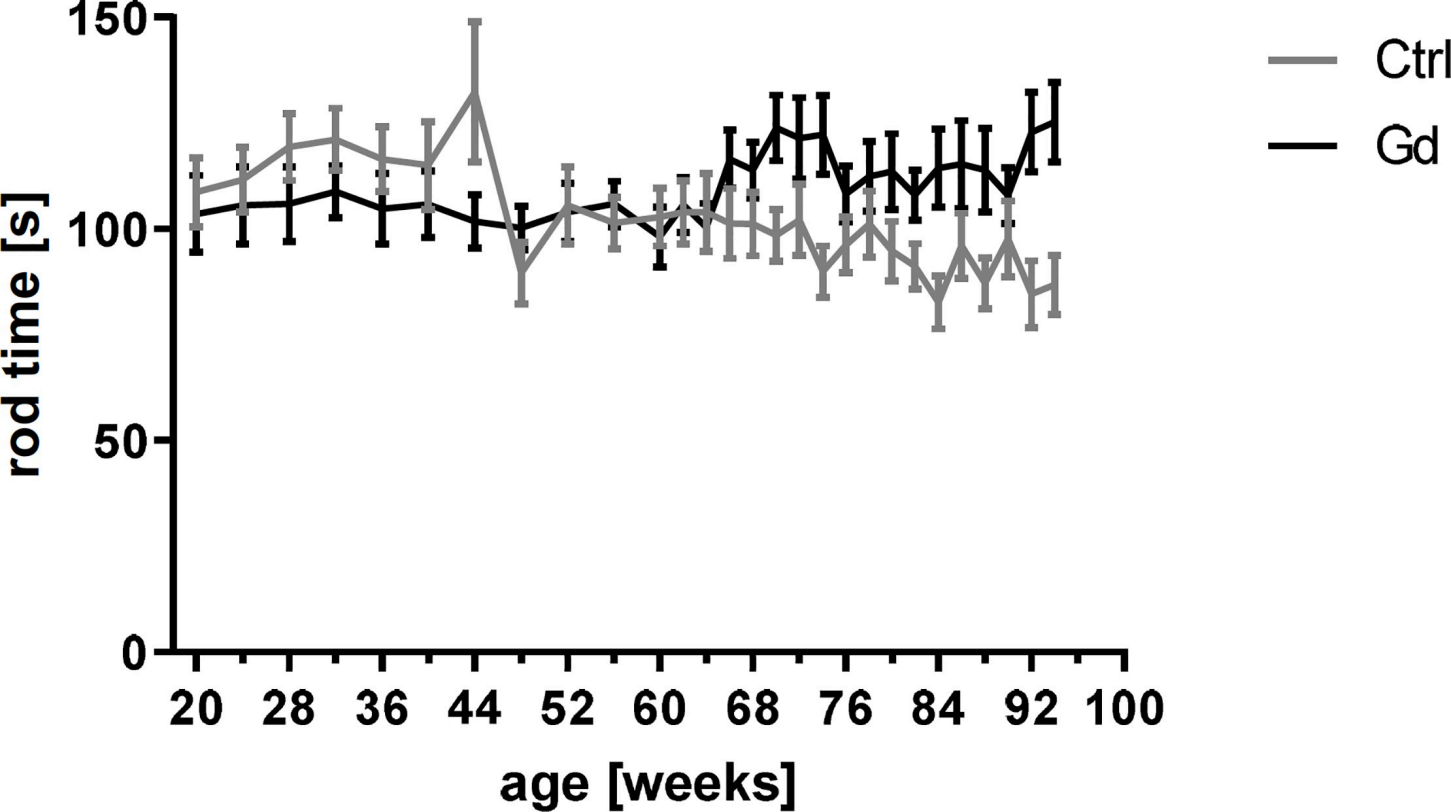
Motor performance. Gd intake had no significant effect on the motor performance phenotype as indicated by the best performance on the rota rod over the study period compared to reference mice. Mean± SEM of 13 or 15 mice per group (mixed-effects analysis, p = 0.1467).

The Gd-intake did not lead to motor impairment, as indicated by the best performance on the rota rod that was not significantly different in Gd exposed and control mice, respectively (Fig 2, p = 0.1467).

**Fig 3:**
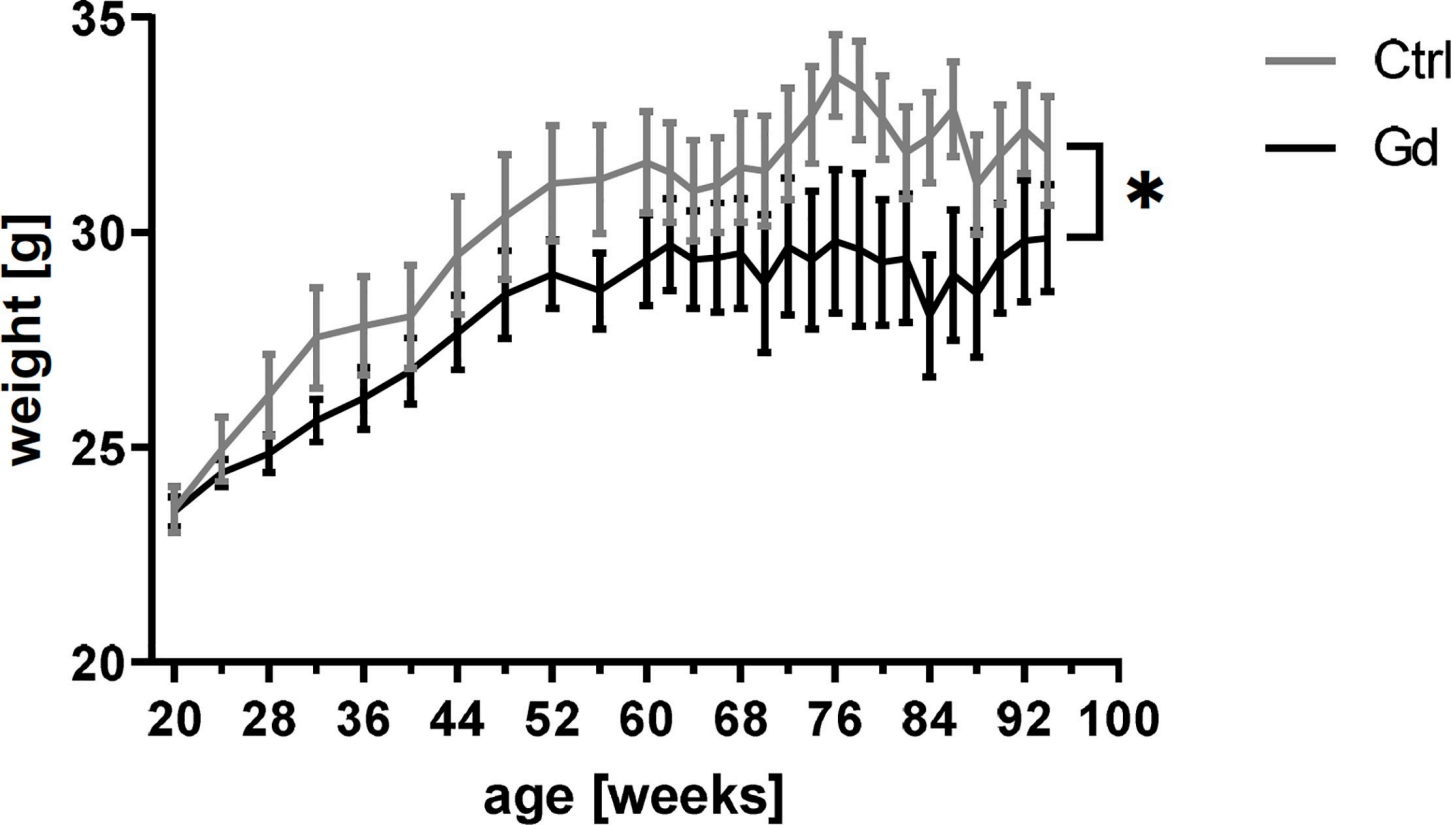
Reduced gain of body weight. Mice orally exposed to Gd showed a significantly decreased gain of body weight over the study period compared to control mice. Mean± SEM of 13 or 15 mice per group (mixed-effects analysis, p = 0.0423).

Regarding the gain of body weight as a marker for general condition, the Gd intake showed a significant effect: starting with an almost directly comparable average weight the Gd exposed group showed a decreased gain of body weight during the study period (Fig 3, p = 0.0423).

**Fig 4:**
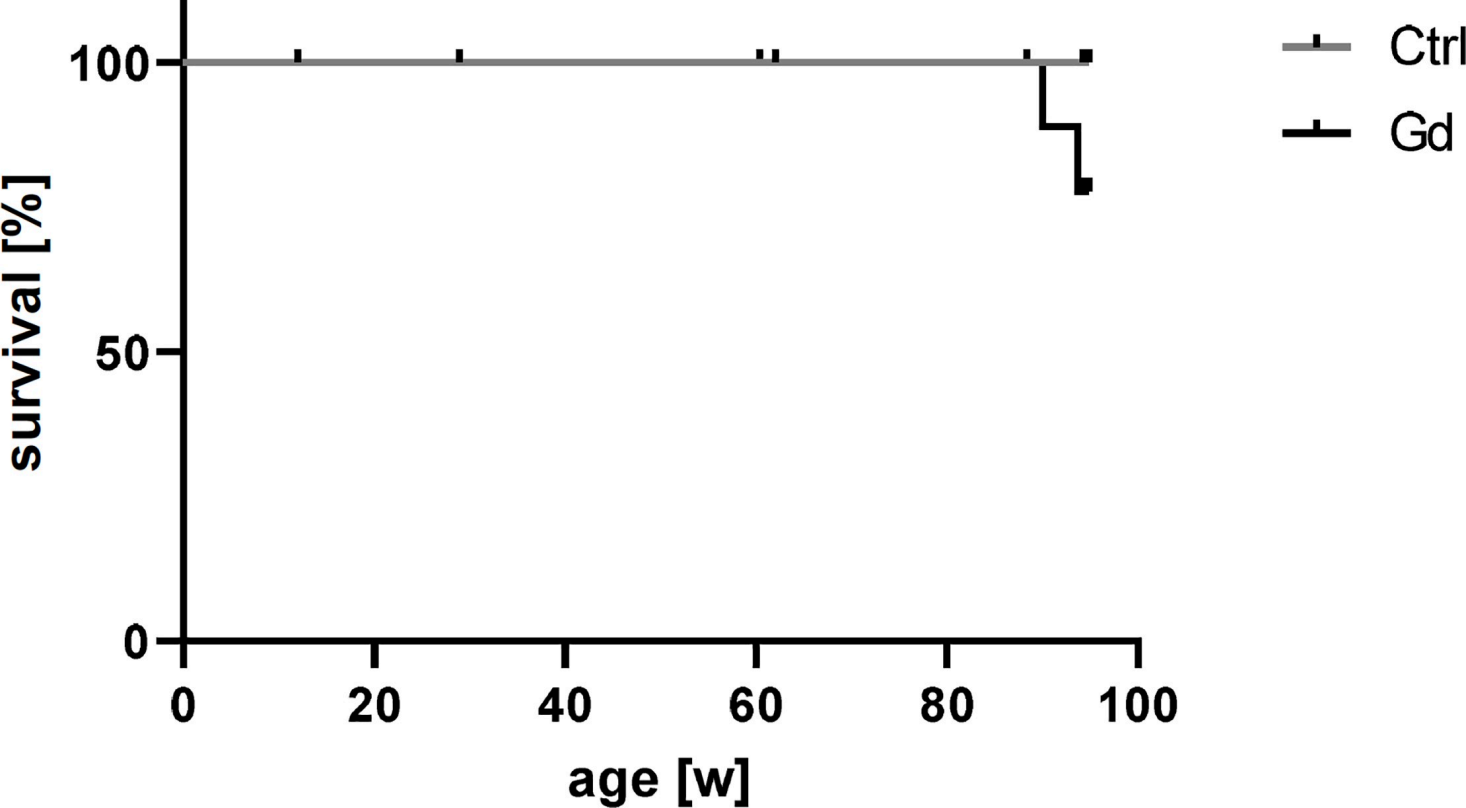
Survival analysis. Oral exposure to Gd did not lead to a significant decrease in survival of treated mice compared to reference mice (N = 13 or 15; Log-Rank (Mantel-Cox) test, p = 0.1451). However, two mice in the Gd-exposed group died spontaneously during the late stage of the study period.

Furthermore, the survival was not significantly affected by the Gd intake (Fig 4, p = 0.1451). However, two mice in the Gd exposed group died spontaneously after 86 weeks and 90 weeks. Although the overall survival of the group was not significantly reduced, this is of note.

### Histopathological findings

**Fig 5:**
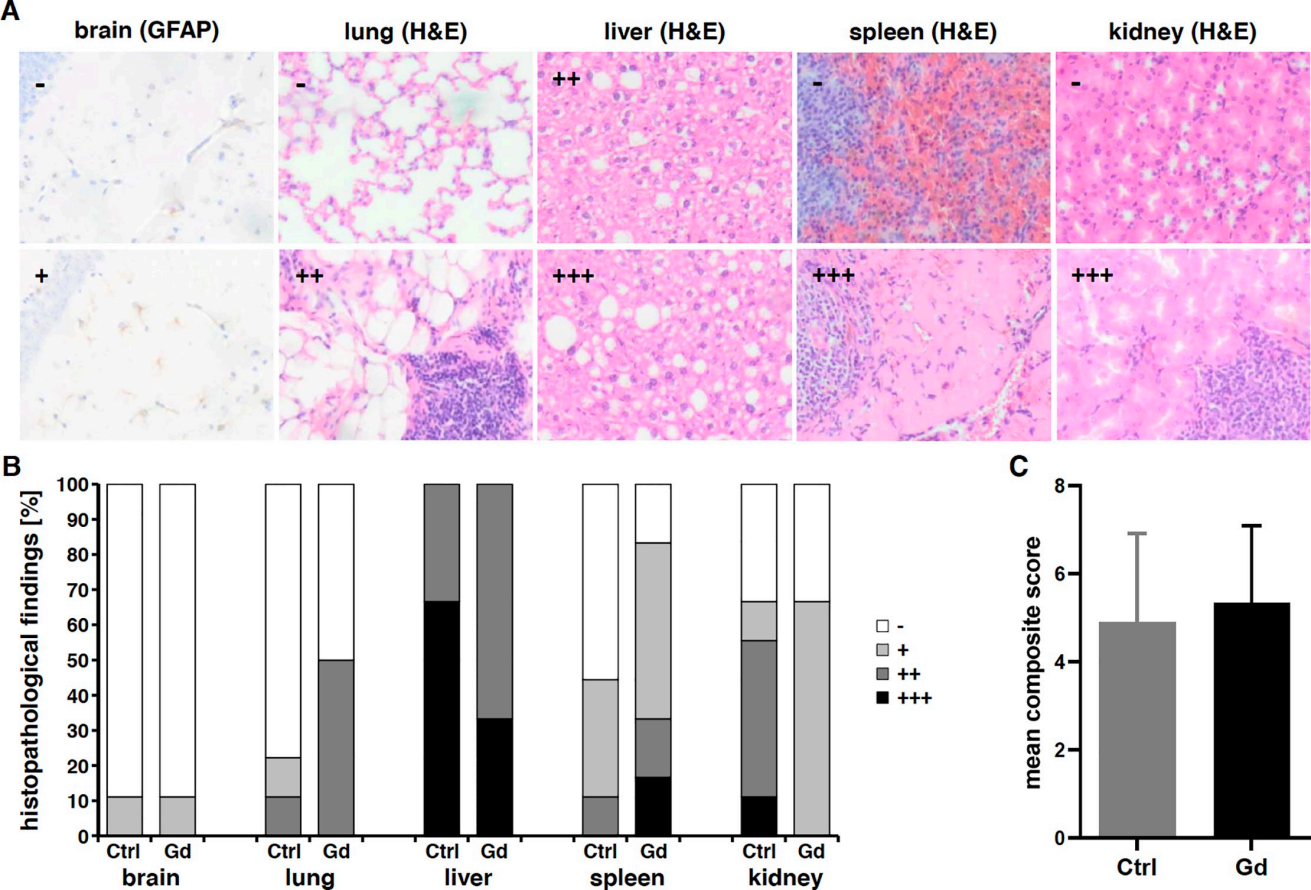
Histopathological findings. Brain as well as peripheral organs such as lung, liver, spleen and kidneys showed pathological alterations to a different extent, absent (-), mild (+), moderate (++), or severe (+++); representative GFAP- and H&E-stainings, (A; see Table 2 for detailed information). All levels could be found in both groups of mice, Gd-exposed and control group, respectively. However, the number of animals without pathological alterations is reduced in Gd-treated mice (B). Analysis of a composite score over all pathological alterations showed no significant difference betwenn Gd-exposed (Gd) and control mice (Ctrl) (C) Mean± SEM of 9 and 6 mice per group, respectively (two-tailed t-test, p = 0.6687).

Histopathological examination revealed no gross signs of cellular alterations or gliosis in the central nervous system of Gd-exposed and control animals as indicated by H&E and GFAP-stainings. Only one animal per group showed mild gliosis in the hippocampus (Fig 5A and 5B). Peripheral organs such as heart and gastrointestinal tract showed no pathological alterations, while cellular infiltrates could be found in lung and kidneys, however to a similar extent in Gd-exposed, as well as control mice. Severe histopathological alterations as indicated by hepatocellular vacuolization due to fatty changes in the liver and a loss of nucleated cells in the red pulp of the spleen could also be found in both groups (Fig 5A and 5B). There were no histopathological alterations within organs of the GI tract in both groups. Overall, fewer mice in the Gd-exposed group showed no histopathological alterations in peripheral organs compared to the control group, but analysis of a composite score over all histopathological alterations revealed no significant differences between both groups (Fig 5B and 5C; two-tailed t-test, p = 0.6687; see Table 2 for detailed individual information).

**Table 2:** Summary of histopathological findings in Gd-treated and control mice (N = 9 and 8, respectively).

	ID	age [w]	Brain	heart	lung	liver	spleen	kidney	GIT
**control**	360	94,3	-	-	-	vvv	-	-	-
	361	94,3	-	-	-	Vvv	-	ii	-
	362	94,3	-	-	-	Vv	c	i	-
	363	94,3	-	-	-	Vv	c vascular pathology	ii	-
	364	94,3	- hemorrhage in 4^th^ ventricle	-	i	Vvv	c	ii	-
	366	94,3	-	-	-	Vvv	-	ii	-
	367	94,3	-	-	-	Vvv	-	-	-
	368	94,3	-	-	-	Vvv	-	-	-
	476	94,9	- mild gliosis (GFAP)	-	ii	Vv	cc	iii	-
**Gd-exposure**	389	93,7	autolytic						
	393	94,3	-	-	- hemorrhage	Vvv	c	i	-
	394	90,0	autolytic						
	395	94,3	-	-	-	Vv	c	i	-
	396	94,3	-	-	-	Vvv	c	-	-
	490	94,9	-	-	ii	Vv	ccc	i	-
	491	94,9	-	-	ii tumor	vv cellular inflitrates	cc	i	-
	493	94,9	- mild gliosis (GFAP)	-	ii	Vv	-	-	-

In general, mice did not show pathological alterations in heart or gastrointestinal tract (GIT). No gross cellular alterations could be found in the CNS, despite two mice (N = 1 within each group) showing mild gliosis in the hippocampus (g). Cellular infiltrates could be found in the lung and kidneys (i). The liver showed hepatocellular vacuolization due to fatty change (v). A loss of nucleated cells could be found in the red pulp of the spleen (c). All pathological alterations could be found to a mild, moderate, or severe extent, respectively (x, xx, xxx). Two mice died before the planned end point and could not be examined because of necrotic changes in nearly all organs due to autolysis.

## Discussion

Our study confirms the presence of low amounts of gadolinium in the brain of healthy mice after chronic oral exposure of Gd containing food pellets without evidence of adverse CNS effects or locomotor impairment. Due to its paramagnetic properties, gadolinium is used in a variety of MRI contrast agents to enhance signal intensity. In order for gadolinium to be used as an MRI contrast agent, it is first bound within a chelating agent to inhibit direct toxicity from the free gadolinium ion. Gadolinium-based contrast agents (GBCAs) are administered intravenously (i.v.). Whereas, i.v. exposure to gadolinium salts in rodents appears to affect the liver [36, 37], i.v. administration of chelated GBCAs additionally affects bone and kidneys [38], outlining differing toxicological properties of the two forms of gadolinium. In terms of the administration route, the distribution and tissue availability of gadolinium from GBCAs administered intravenously differs markedly from those of gadolinium taken up after oral exposure. Chelating the gadolinium ion using multidentate organic ligands significantly reduces the interaction of the metal ion with the biological system, thereby dramatically decreasing the associated risk for gadolinium toxicity. To this point, the bone and kidneys appear to be the target organ of i.v. GBCA administration in rodents with normal renal function [38], while the liver appears to be primarily affected by oral exposures to gadolinium salts [39–41]. This is mainly because Gd is expected to be poorly absorbed through the gastrointestinal (GI) tract [42]. Therefore, it is expected that the bioavailability of gadolinium for uptake in the small intestine will be very limited and is, per se, not truly comparable to i.v. dosages. Once orally absorbed, Gd was shown to be deposited in bone, liver, kidney, and lungs [42]. For gadolinium trichloride, a water-soluble gadolinium compound, gadolinium accumulation was measured in different organs of rats repeatedly exposed via oral gavage to doses up to 1000 mg/kg/day for 28 days in total [40]. Gadolinium was reported to be accumulated in the liver, kidney, spleen and bone in a dose dependent manner. In accordance to our study, histopathological assessment showed no obvious toxicity to liver, kidney, spleen, lung, blood, and heart by oral administration, however, the potential brain accumulation after oral exposure was not assessed.

Gd related toxicity following i.v. exposure has been recognized for at least 10 years, with the initially described condition being nephrogenic systemic fibrosis (NSF) [43, 44]. In several recent studies, Gd was shown to accumulate in the brain of mice and humans after i.v. application of GBCAs irrespective of renal function and GBCAs stability class [9, 13, 20, 28]. Despite the increasing evidence of Gd accumulation in the brain following i.v. GBCA administration [11, 16, 25, 28, 45], knowledge about potential Gd associated CNS effects including locomotor alterations is very limited both in the animal model and in humans. In a recent study, Gd was retained in the brain of mice during postnatal development following GBCA administration to pregnant mice [46]. Due to perinatal exposure, the retained Gd was suspected to lead to impaired brain development in mice [46]. To date, no other recent study assessing Gd accumulation in the brain has been associated with any severe clinical symptoms or adverse health effects, especially in regard to brain toxicity. There is currently no clear evidence on mechanisms, by which Gd may affect brain tissue (e.g. protein synthesis, axonal transport, and neurotransmitter-related events).

Previous studies reported Gd levels of 0.2–4.0 nmol/ g brain tissue in healthy rats after repeated intravenous administration of linear GBCAs, resulting from a supraclinical dosing regimen [16]. In the clinical setting, Gd levels in postmortem human brain specimens are typically similar (up to 0.1–6.0 nmol/ g brain tissue) across a range of linear and macrocyclic GBCAs [11, 28, 45]. A recent study assessing brain accumulation of Gd and its partial clearance in rats after 20 weeks, did not detect any treatment-related histopathologic findings in rats over a study period of 20 weeks; the accumulation dose varied between 1.39± 0.20 nmol/ g brain tissue vs. 2.49± 0.30 nmol/ g brain tissue (mean± SD) dependent on the administered dose and was partially cleared in healthy rat brains up to 50% after 20 weeks, respectively [47]. Our study was performed to evaluate the long-term effects of Gd brain accumulation and potential effects of this metal on general condition, weight loss, motor performance, as well as survival in primarily healthy mice. We administered Gd-chloride via food pellets resulting in a daily intake of approximately 0.57 mmol/ kg body weight over a study period of 90 weeks in total to observe long-term effects of potential Gd associated toxicity over the almost entire lifespan of the investigated mice. Despite its low intracerebral accumulation, which cannot be fully compared to the i.v. administration studies above, the chronic oral intake of Gd did not result in significant adverse CNS effects on motor performance in our study. In contrast, there might be an impact on general health status, as indicated by a significantly decreased gain of body weight, however, histopathological examination revealed no significant cellular tissue alterations in peripheral organs including the gastrointestinal tract of Gd-exposed and control animals, respectively.

Interestingly, a prior study conducted by Haley et. al. [41] provides evidence for mild tissue changes in the spleen and liver after chronic oral Gd exposure. The only significant exposure related changes were perinuclear vacuolization of the parenchymal cells of the liver (which were found in both groups of our study cohort) and a coarse granularity of the their cytoplasm in rats exposed to 500 mg/kg-day body weight gadolinium chloride. Additionally, the most relevant study yielding information on distribution and accumulation after oral exposure is the repeated dose toxicity study for gadolinium trichloride in rats [40]. The effects of oral exposure of animals to Gd were additionally evaluated in another short-term-duration study by Ogawa et al. [39]. Here, Gadolinium exposure at 423 mg/kg-day resulted in concordance to our study in decreased body weight gain in both males and females and microscopic lesions were observed in the forestomach and submucosa of the stomach, consisting of hyperkeratosis and eosinophil infiltration, respectively. Similar findings were evaluated by Barnhart et al. [48] who intravenously injected gadolinium trichloride (at a dose of 100 μmol/kg, i.e. 26.36 mg/kg) in rats. After injection, gadolinium was found primarily in liver and spleen. The results of the study suggest that after gadolinium trichloride was injected it was subject to complexation with proteins and colloid formation. The biodistribution of gadolinium trichloride was hence mostly depending on uptake of the colloids by phagocytic cells of the reticuloendothelial system and consequent storage of the material in organs that are part of this system, such as liver and spleen. Although we did not assess Gd concentrations in peripheral organs, significant differences in tissue alterations were absent on histopathology between both groups, especially the GI tract did not show any remarkable tissue alterations. Of note, no prior study did focus on Gd brain accumulation and its potential adverse CNS effects after oral administration.

Although the oral exposure to Gd chloride resulted in low, but significant brain accumulation in healthy mice in our study group (up to 0.033± 0.009 nmol Gd/ g brain tissue) compared to the control group (0.006± 0.001 nmol Gd/ g brain tissue) we found no clinically visible adverse CNS effects (e.g. paralysis, seizures), impairment in locomotor activity, or gross cellular alterations in microglial activity (after GFAP stainings) in the brain of mice between both groups.

Further, although the survival of the Gd-treated group was not significantly different compared to the control group, two Gd-treated mice died spontaneously during the study even if relatively late in regard to the overall lifespan of mice (90.0 and 93.7 weeks of age, respectively). The lack of adverse CNS findings in the long-term setting of our study is in agreement with findings in a previous study in rats [47]. However, the levels of Gd/ g brain tissue in our study were markedly lower than those in previous rat and postmortem human studies after repeated i.v.-administration of GBCAs [11, 16, 28, 45, 47] and after partial clearance of Gd as assessed in rats after a time course of 20 weeks after repeated i.v.-administration of GBCAs [47].

It is worth mentioning, that the uptake and distribution of intravenously administered GBCAs differs substantially from those observed after oral exposure to gadolinium salts. In contrast to prior studies, our study includes a chronic exposure to Gd via food pellets during the almost entire life span of mice with proof of substantial brain accumulation and absence of adverse CNS effects or changes in locomotor abilities, even though Gd exposed mice show mild adverse health effects in the periphery as indicated by a less increase in body weight. These observation strengthens the assumption that substantial Gd accumulation within the body might not necessarily be related to CNS effects, even though the brain concentrations of Gd were fairly low. The assessment of adverse health effects due to a chronic oral exposure of Gd is especially highly relevant since Gd represents an emerging environmental contaminant due to the increasing use of GBCAs in medical imaging [29, 30], as there is still relatively little knowledge of the biogeochemical or anthropogenic cycles of Gd in the environment. Due to their high stability, Gd complexes (such as Gd-(DTPA)) were shown not be sufficiently removed by commonly used wastewater treatment technologies [30]. Although the measured concentrations of anthropogenic Gd (shown for concentrations in San Francisco Bay [30]) were well below the threshold of ecotoxicological effects, the increasing presence of anthropogenic Gd as environmental contaminant cannot be disregarded. Our study may contribute to a better understanding of potential adverse health effects after long-term oral exposure (e.g. due to uptake of contaminated water) without detectable CNS effects.

### Limitations

Our study has several limitations that need to be taken into account when interpreting the data. First, the measured intracerebral Gd concentrations were comparatively low, since prior studies in rats and postmortem human studies after repeated intravenous GBCA injections reported higher intracerebral doses. Second, methodological limitations are present since no toxicological end points and peripheral Gd concentrations were evaluated, making the adverse significance of the decreased body weight gain difficult to interpret. Third, the distribution, bioavailability and potential harmful effects of Gd depend substantially on the route of administration, and the chemical form of Gd. Thus, the oral application of Gd as a chloride-salt cannot be fully compared to the intravenous injection of chelated Gd. Furthermore, our study did not investigate the location of Gd in the brain of mice. We measured Gd levels in the entire left cerebral hemisphere of each animal, which may underestimate potential peak concentrations, e.g. in the dentate nucleus [13, 14, 18, 20, 28]. Several recent studies did investigate local Gd distributions within the brain and the colocalization of Gd with other elements using ICP-MS. Importantly, these studies did not find obvious alterations of brain tissues which is in accordance with our study [49–51]. Additionally, our study did not specifically assess neurotoxicity based on histopathology by using dedicated immunostainings. Although we did not detect any adverse CNS effects or gross cellular alterations of brain tissue in healthy mice, this can certainly not be automatically assumed to hold true for all species, including humans, or for subjects with underlying disorders. Last, the limited life-time of rodents is most likely not suitable to simulate all long-term toxicology effects in humans.

Further studies are required to explore whether a higher oral dose of administered Gd could induce adverse CNS effects or whether such effects may potentially occur in subjects with underlying diseases or genetic predispositions. Results should be supported by a further evaluation of biochemical parameters, including bioavailability and clearance of Gd at various dosage levels and for various exposure times. Whether higher doses of Gd potentially affect important biochemical pathways in the CNS, including enzymes or protein synthesis, remains to be elucidated.

## Conclusion

Although the absorption and bioavailability of gadolinium after oral administration via food pellets is expected to be very limited, it accumulates in the brain of mice after chronic oral exposure to a low but significant amount. Our prospective study suggests that low levels of intracerebral Gd do not lead to detectable CNS effects, and in particular do not impair locomotor abilities in the healthy murine model, even over a very long exposure time. Gd exposure had mild adverse effects on gain of body weight, however, survival was not affected following life-long exposure in mice.

## Supporting information

S1 Raw data (XLSX)

S2 Raw data (XLSX)

S3 Raw data (XLSX)
